# Impact of anesthetist licensing examination on quality of education in Ethiopia: a qualitative study of faculty and student perceptions

**DOI:** 10.1186/s12909-023-04452-5

**Published:** 2023-06-22

**Authors:** Yohannes Molla Asemu, Tegbar Yigzaw, Firew Ayalew Desta, Tewodros Abebaw Melese, Leulayehu Akalu Gemeda, Fedde Scheele, Thomas van den Akker

**Affiliations:** 1 Health Workforce Improvement Program (HWIP), Jhpiego, an affiliate of Johns Hopkins University, Ethiopia country office, Addis Ababa, Ethiopia; 2 Athena Institute, Faculty of Science, Vrije Universiteit Amsterdam, Amsterdam, the Netherlands; 3 Health Professionals’ Competency Assessment and Licensing Directorate, Ethiopian Ministry of Health, Addis Ababa, Ethiopia; 4 Ethiopian Association of Anesthetists (EAA), Addis Ababa, Ethiopia; 5 Department of Anesthesia, Addis Ababa University, Addis Ababa, Ethiopia; 6 OLVG Teaching Hospital, Amsterdam, the Netherlands; 7 Amsterdam UMC, Amsterdam, the Netherlands; 8 Chair Legislative College for Accreditation of Residency Training, Dutch Royal Medical Council, Utrecht, 2016-2019 the Netherlands; 9 Department of Obstetrics and Gynaecology, Leiden University Medical Centre, Leiden, the Netherlands

**Keywords:** Anesthesia, Non-physician anesthetists, Associate clinician anesthetists, Education Quality, National Licensing Examination, Assessment

## Abstract

**Background:**

Ethiopia drastically increased the anesthesia workforce density by training *‘associate clinician anesthetists’* as a task-shifting and sharing strategy. However, there were growing concerns about educational quality and patient safety. Accordingly, the Ministry of Health introduced the anesthetist national licensing examination (NLE) to assure the quality of education. However, empirical evidence is scarce to support or refute the overall impact of NLEs, which are relatively costly for low- and middle-income settings. Therefore, this study aimed to explore the impact of introducing NLE on anesthetists’ education in Ethiopia.

**Methods:**

We conducted a qualitative study using a constructivist grounded theory approach. Data were prospectively collected from ten anesthetist teaching institutions. Fifteen in-depth interviews were conducted with instructors and academic leaders, and six focus groups were held with students and recently tested anesthetists. Additional data were gathered by analyzing relevant documents, including versions of curricula, academic committee minutes, program quality review reports, and faculty appraisal reports. Interviews and group discussions were audiotaped, transcribed verbatim and analyzed using Atlas.ti 9 software.

**Results:**

Both faculty and students demonstrated positive attitudes toward the NLE. Student motivation, faculty performance, and curriculum strengthening were the three primary changes that emerged, resulting in three subsequent spin-offs on assessment, learning, and quality management practices. Academic leaders’ dedication to evaluating examination data and turning these into action led to changes that improved education quality. Increased accountability, engagement, and collaboration were the predominant factors facilitating change.

**Conclusion:**

Our study indicates that the Ethiopian NLE has prompted anesthesia teaching institutions to improve their teaching, learning, and assessment practices. However, more work is required to improve exam acceptability among stakeholders and drive broader changes.

**Supplementary Information:**

The online version contains supplementary material available at 10.1186/s12909-023-04452-5.

## Introduction

The United Nations adopted the 17 integrated Sustainable Development Goals (SDGs) in 2015, calling on member states to act to eradicate poverty and health inequities by 2030. The health targets of the SDGs, such as improving access to safe surgical care, will only be met with transformational reforms to the health workforce [1, 2]. Task shifting and sharing—transferring, delegating, or sharing specific tasks with existing or newly formed health cadres with shorter training and fewer credentials—can improve the efficiency of the health workforce and access to essential health services, including surgery and anesthesia. Task shifting and sharing strategies are particularly important in low- and middle-income countries (LMICs), where the lack of medical doctors limits access to safe surgical care [2–5]. Through this strategy, Ethiopia increased the anesthesia workforce by training *‘associate clinician anesthetists*,*’* a term we introduced in this article to refer to the group of non-physician anesthetists (also called anesthetists) who can independently provide a wide range of anesthetic care. The training models (Table 1) include a one-year vocational training for diploma nurses, a 3-year bachelor’s degree training for diploma nurses, a 4-year bachelor’s degree training for high school graduates, a 2-year post-bachelor master’s qualification training, and a 4-year post-master’s PhD training [6–9].

**Table 1:** Training models/ pathways for anesthetists in Ethiopia

Entry behavior	Duration of training	Qualification earned
Diploma nurses with 2 years of clinical experience	One year (vocational training)	Level 5 anesthetic nurse
Diploma nurses with 2 years of clinical experience OR level 5 anesthetic nurse	Three years (university/ college training)	Bachelor’s degree in Anesthesia
High school graduates	Four Years (university training)	Bachelor’s degree in Anesthesia
Bachelor’s degree in Anesthesia (from both backgrounds as a nurse or high school graduate)	Two years (university training)	Master’s degree in Anesthesia
Master’s degree in Anesthesia	Four years (university training)	PhD in Anesthesia

A typical curriculum for the baccalaureate degree program is composed of first-year biomedical courses, a year of core clerkship courses (Surgery, Gynecology and Obstetrics, Internal medicine, and Pediatrics), followed by two years of anesthesia specialty courses (known as senior year studies), with public health courses running in parallel from year 1 to the end. The second-year core clerkship courses are exempt for those with diploma nursing backgrounds.

Between 2012 and 2018, the number of anesthetist teaching institutions increased tenfold, and annual enrollment increased 12-fold. However, this rapid expansion stretched capacity, resulting in difficulty maintaining quality. Senior institutions were impacted by growing enrollments and the transfer of some of their experienced faculty to newer institutions. Newly established institutions were also challenged with predominantly junior faculty, frequent staff attrition, inadequate teaching resources, and limited clinical practice sites, which hamper learning experiences [8, 9]. As a result, there was a growing concern that the rapid changes and variability in the curriculum implementation might compromise the quality of education and competence of graduates [7, 9]. At the same time, medico-legal reports showed a substantial increase in patient safety incidents attributable to anesthesia practice [10]. These issues increased the demand for external quality assurance and regulation.

The effectiveness of workforce development strategies depends on national frameworks regulating demand and supply issues. Failure to regulate could endanger the public by allowing incompetent graduates to practice [2, 11, 12]. Therefore, a critical agenda item for healthcare regulators is guaranteeing a minimum level of competence [13]. Regulation can occur at different levels, including self-regulation, institutional-level regulation, and statutory restrictions imposed by governmental authority at the state or national level. In this regard, accreditation and licensing are the main state-level mechanisms to regulate healthcare education and practice [12, 13]. Public and private authorities should collaborate to ensure that all health workers, including those with new roles through task sharing and shifting, provide care of sufficient quality [2].

In line with this, the Ethiopian Ministry of Health (MOH) prioritized health workforce regulation in its 10-year Human Resources for Health strategic plan [14] and consequently introduced a national licensing examination (NLE) for 13 baccalaureate health cadres, including anesthetists [8, 15]. Following a pilot program between 2015 and 2018, the MOH mandated the NLE as a requirement for licensing newly graduated professionals in 2019. The current single-step, paper-and-pencil anesthetist NLE is designed based on a task analysis study [7] and aimed at assessing the application of scientific knowledge through a 200-item context-based multiple-choice question format. The exam covers five main essential competency areas: patient care, professionalism, research, leadership and management, and health promotion and disease prevention. In 2019, the MOH pilot-tested an Objective Structured Clinical Examination (OSCE) for anesthetists as a learning experience (i.e., OSCE results were not used to establish pass or fail decisions on the licensing exam). To date, more than 100,000 health professionals, including thousands of anesthetists, have sat for the NLE.

NLEs aim to ensure a minimum competence level to start safe clinical practice. By setting up a system that identifies less competent graduates, NLEs are believed to ensure patient safety and positive outcomes [16, 17]. Some studies have indicated that the quality of education [18–20], competence of graduates [16, 21], and patient outcomes [22, 23] can be assured through the effective implementation of NLEs. Opponents of NLEs, on the contrary, raise the untoward effects of restricting innovation and creativity by narrowing the focus of curricula [24, 25]. Others argue that program accreditation and other forms of assessment are better alternatives [24, 26, 27]. Supporting or refuting either claim requires gathering scientific evidence, known as “validation” [28, 29].

Given that the effect of exams in improving learning outcomes should outweigh the associated costs, data on exam consequences constitute the most fundamental source of validation evidence [30]. Like any diagnostic test, the impact of educational exams on students, faculty, and the larger academic environment forms the basis of evidence for their use [30–32]. However, compared to other validation sources, accumulating consequence validity evidence is challenging as the concept has not been extensively discussed in traditional notions of validity, making it difficult for educators to understand [30]. As a result, there is a lack of adequate empirical evidence, especially from LMICs, to support or refute the impact of NLEs on the training and competence of students and overall health outcomes [12, 21, 26].

This study aimed to explore the educational impact of the Ethiopian Anesthetist National Licensing Examination on the anesthesia training program and develop a theory that may explain related change processes.

Specifically, the study questions explored in this research were:

1) What are the experiences and perspectives of anesthesia program academics, students, and graduates regarding the introduction of NLE?

2) How do anesthesia programs modify their learning and assessment practices due to the introduction of the NLE?

## Methods

### Study design

We employed qualitative methodology based on a constructivist grounded theory approach. The research process involved collecting data through interviews, focus groups, and document reviews. The Preservice Education conceptual model developed by Johnson et al. was used to frame our study [33]. The COnsolidated criteria for REporting Qualitative research (COREQ) was used to frame the overall research process and ensure the study’s rigor and comprehensiveness of reporting [34]. Investigators regularly refer to the 32-item checklist from study conceptualization and proposal writeup to manuscript review and finalization. In particular, the first and third investigators (YMA and FAD) regularly check adherence to each checklist step throughout the study and bring any concerns to the regular team debriefings. A complete checklist appraised by investigators is attached as a supplement (supplement 1).

### Sample and sampling procedure

Purposeful sampling was employed to select a total of 46 study participants for in-depth interviews and focus groups (FGs) from the ten teaching institutions that have had graduate anesthetists since the beginning of the NLE. Fifteen in-depth interviews were conducted with 13 male and two female full-time academics who had at least four years of teaching experience at their current institution (i.e., worked there since the start of NLE). All departments involved in the training of anesthesia students, including basic sciences, public health and clinical specialties including anesthesia were represented. The higher number of male participants was because anesthesia instructors in Ethiopian teaching institutions are predominantly men (Table 2).

**Table 2:** Demographic characteristics of interviewees (n = 15) and FG (n = 31) participants

Interview participants (n = 15)			Focus group participants (n = 31)		
Characteristics		**Total**	**Characteristics**		**Total**
Gender	Male	13	Group of participants	Students	15
	Female	2		Graduates	16
Year of experience	4–6	7	Students by gender (n = 15)	Male	7
	7–9	6		Female	8
	10–12	1	Graduates by gender (n = 16)	Male	7
	> 12	1		Female	9
Position held	Program instructor	7	Duration in year since taking the NLE (for graduates; n = 16)	< 1	10
	Program head	4		1–2	4
	Higher-level academic leader	4		> 2	2

Note: Program heads and higher-level academic leaders were also engaged in teaching anesthesia students

On the other hand, three FGDs were conducted with 15 final-year students (8 females) and three more with 16 recent graduates (9 females). All study participants were chosen based on the desired essential theoretical characteristics, including gender, current roles, prior experiences, geographical location and generation of institutions.

### Participant recruitment

#### In-depth interviews

The principal investigator (YMA) phoned and emailed eligible participants to invite them to a face-to-face discussion about the study. The principal investigator (PI) or trained data collector then met with each volunteer study participant to explain the study procedures and obtain written informed consent for participation. Interviews were arranged based on the participants’ schedules.

### Focus groups

Using contact information obtained from respective programs, the PI phoned and emailed potential participants to invite them to a virtual discussion about the study. Those who agreed to participate in the study received an invitation and a study information sheet by email. Each participant signed a consent form upon arrival for the group discussion.

### Data collection

Interview and focus group guides were constructed based on the seven main standard areas in the national anesthesia education standards [35], which were adapted from the World Federation for Medical Education (WFME) basic medical education standards [36]. These areas were (1) curriculum, (2) assessment, (3) students, (4) academic staff, (5) educational resources, (6) quality assurance, and (7) governance and administration. Each guide contains 11 items to capture a range of constructs (supplements 2 and 3). The authors subjectively evaluated the presentation and relevance of these tools and translated them into Amharic, the local language. Interview guide was pilot-tested with two volunteers.

The PI and six data collectors with at least a master’s degree and prior experience in higher education teaching and similar qualitative research conducted interviews and FGs in Amharic using semi-structured guides. All underwent a one-day intensive training that included mock sessions and feedback on note-taking, ethical issues, and probing techniques.

Each interview and focus group started with basic questions regarding participants’ own perceptions of the NLE, then moved on to their observations and experiences about changes to the anesthesia program as a direct result of the NLE. Probing questions were used to elicit additional data and clarify information.

Twelve face-to-face interviews were conducted by trained data collectors between September 20 and 24, 2021, and the remaining three between October 5 and 10, 2021 by the PI. The PI spent additional time at three study sites to thoroughly understand the phenomenon. No further data was collected after this point, as no new information emerged during coding and saturation was reached. Moreover, between September 23 and 25, 2021, a total of six face-to-face focus groups (3 with students and 3 with graduates) were held in Addis Ababa, with each group’s members carefully assembled based on the essential characteristics of gender, relevant prior experience, geographical location and university generation. The interviews lasted between 33 and 96 min (mean 56 min), while the focus groups lasted between 73 and 122 min (mean 94 min). All interviews and focus groups were recorded using digital voice recorders (Sony ICD-PX370).

In addition, secondary data were systematically gathered from program self-review reports (2017–2020), action plans, curricula, nine randomly picked department academic committee minutes from each institution (2018–2020), three-year performance appraisal reports of selected three instructors from each institution, and previously used learning and assessment tools. Secondary data were gathered from eight of the ten institutions, as two were deemed unsafe for travel due to political instability in the northern part of the country.

### Data analysis

Audio-recorded interviews and focus groups were transcribed verbatim and checked for transcription accuracy. The PI distributed interview transcripts to all respondents for verification, with eight making additions and clarifications, six agreeing, and one not responding. The PI coded and analyzed the interview and FG transcripts separately. Key subthemes and themes were derived from the transcripts using an inductive approach until no new themes emerged. Regular debriefings were held between the lead and the second investigators (YMA and TY) to ensure accurate data coding, analysis, and interpretation.

Through a continuous iterative process, information on transcripts was coded and thematized into key categories explaining the underlying theoretical construct. The coding mainly consisted of Initial, Process, Focused, Axial, and Theoretical Coding. Initial coding was done by carefully reading transcripts line by line and developing tentative notions. By breaking down the bulk qualitative data into pieces, the PI thoroughly understood the data content. Simultaneously, process coding was applied to demonstrate the link between actions and consequences. As the data analysis progressed, the frequent codes were selected and refined to distill concepts into sub-themes. Axial coding was then employed to reorganize subthemes and themes by identifying the most relevant codes. Finally, theoretical coding was used to unearth the main category connecting all groups generated thus far and develop the explanatory model. Analytic memos were also created and utilized alongside to facilitate theory generation.

As a result, a theory was formulated based on the themes and concepts that emerged throughout the process. By selecting the two main categories of (a) the licensing exam as a change agent and (b) the change driving forces, a substantive theory was developed by hierarchically organizing sub-categories and concepts to illustrate interrelationships and causality. As applied to constructivist grounded theory research, the boundaries within the categories and sub-categories should be viewed holistically, as each one is different but not necessarily exclusive of the others. The analysis output and selected English translations of participant quotes are presented using texts, tables and matrix. Data from the secondary document review is also discussed and displayed as applied. Atlas.ti version 9 software was used to facilitate data analysis.

### Ethical considerations

We obtained ethical approval for the study from the Johns Hopkins Bloomberg School of Public Health’s (JHBSPH) Institutional Review Board (IRB # 17,778). Local ethical approval was secured from the Ethiopian Public Health Institute (EPHI-IRB-380-2021). Accordingly, we strictly followed the JHBSPH Responsible Conduct of Research and the Ethiopian National Research Ethics Review Guidelines as guiding frameworks throughout the various phases of the study. We obtained support letters from the ministries of health and education. Institutional approval letters were also secured from the respective deans. Permissions to conduct the study were attained from the Ministry of Health (MOH), Ministry of Science and Higher Education (MOSHE) and respective leaders of teaching institutions. Informed written consent was obtained from all study participants, and measures were taken to protect autonomy and data confidentiality.

### Authors

A total of seven investigators (all men) were involved in the study. Three of them (TA, FS, and TY) are physicians with a PhD and extensive expertise, including in qualitative research. The other four have backgrounds in anesthesia (YMA, LAG), nursing (TAM), and statistics (FAD). The third researcher (FAD) also has a PhD in public health and rich experience in monitoring, evaluation, and research. The first two researchers (YMA, TY) are educators and active members of a technical working group that has been supporting the Ministry of Health in introducing and implementing the NLE, while the other two are leaders of the licensing body (TAM) and the Ethiopian Association of Anesthetists (LAG). The first researcher (YMA) is a PhD candidate in the field of medical education and received a fellowship and postgraduate certificates in health professions education and regulation from FAIMER and Keele University, respectively. The three (TA, FS, and TY) are his supervisors.

## Results

**Fig. 1:**
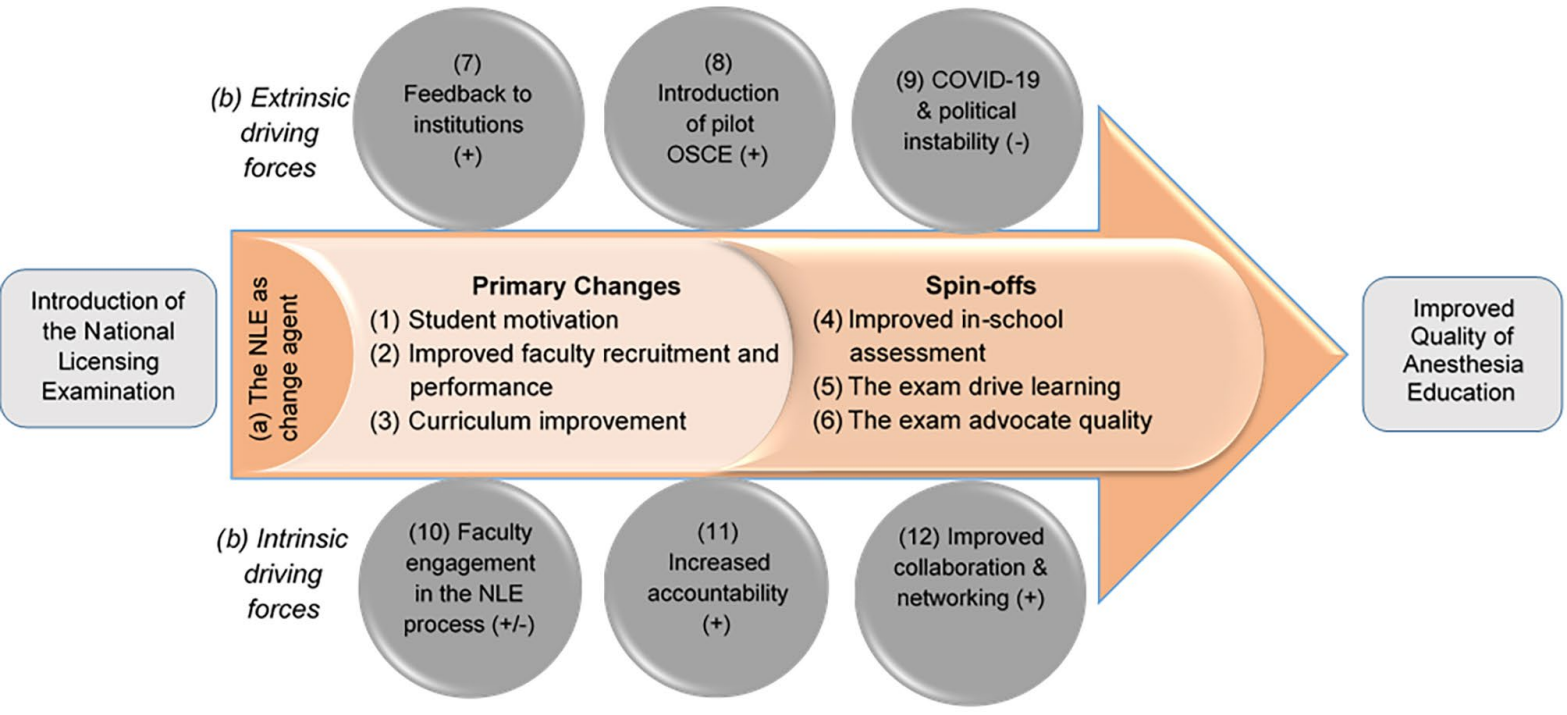
Effect model explaining the overall change process in anesthesia education as a direct result of the NLE *Note: Symbols: +, Positive driving force; -, Negative driving force; +/-, Both positive & negative driving effects*

Interview and focus group transcript analysis yielded two central categories (a and b) and twelve associated sub-categories (1–12). The twelve sub-categories that revealed the perceptions of academics and students towards educational changes (1–6) and the driving forces for changes (7–12) were then combined into a framework for understanding how the NLE affects anesthesia education (Fig. 1).

Changes made to the quality of anesthesia education as a direct result of the NLE were influenced by the academic leaders’ commitment to translating feedback into action by increasing accountability and follow-up on program heads, instructors, and students, thereby improving their engagement and collaboration within and outside their teaching institutions. The six prominent changes will be explored thoroughly, followed by a summary of the six driving forces.

### The licensing exam as a change agent

Three primary changes related to students, faculty, and curriculum have emerged, along with three spin-offs on assessment, learning, and quality assurance. Five of these changes emerged as common themes in both the FG and interview transcript analyses, while the sixth, on *quality assurance*, emerged in the interview transcript only.

### The exam as a student motivator

Nearly all respondents emphasized the positive behavioral changes observed in students. Raising program expectations by setting higher NLE pass rate targets and regularly reminding students of the need to pass the NLE for employment were seen as behavior drivers. Although this expectation was largely communicated with senior-year students, it was said to improve student interactions, teamwork, dedication to the profession, time spent reading additional resources, and classroom attendance.

Student 08: *With the licensing examination in mind, we now ask each other case-based questions everywhere, including classrooms and dorms… We use our cell phones to refer to learning documents, ask questions, and discuss things while on duty in the operating room… we used to study only when we were on campus but now we studied even during school break.*

Programs reported an increase in the number of qualified students applying to join the program. Some interviewees claimed that the higher pass rate among anesthesia students attracted better-prepared students to the program, although focus group participants argued that they were only aware of the NLE in their third year. Programs also described improved student support primarily targeting senior-year students, those with the lowest in-school grades, and those taking courses where the previous cohort had lower NLE scores.

### The exam improved faculty recruitment and performance

Most programs reported improvements in faculty enrollment and assignments, with the exam serving as a justification for hiring more academic and technical staff. Interviewees from better-performing programs reported increased faculty engagement in various administrative posts, with anesthesia faculty accounting for more than half of the academic council at one institution. Most FG participants also reported positive faculty behavioral changes, including improved commitment to extra effort in preparing students for the NLE, preparation for and participation in academic discussions, interaction with students, complaint handling, and commitment to learning.

Graduate 02: *Our morning sessions, seminars, and other academic discussions are well-attended and supported by most of our instructors. If you made a mistake in the operating room in the past, you would be told to leave the room right away… But now, there are active discussions and arguments with teachers everywhere, including in operating rooms. Insults and expulsions are now very rare.*

Faculty appraisal reports showed overall improvement in the performance of professional course instructors, with professionalism competency showing the most improvement, followed by student assessment and feedback. However, quite a few respondents argued that these changes are limited to instructors who teach courses that the NLE directly assesses.

### The exam stimulated curriculum improvement

The majority of FG and interview respondents perceived that low NLE performance scores forced programs to revise program focus, learning contents, and delivery approaches.

Instructor 04: *The licensing exam tells us which areas we should concentrate on. The exam served as a blueprint because anesthesia is such a broad field, and as they said, “he who chases ten cannot catch one.“ It also helped us address overlooked areas by adding key competencies. For instance, we strengthened the teaching and assessment of postoperative care … and ICU … by extending the course duration from two months to two full semesters [8 months]. The newly redesigned integrated national curriculum reflected these changes as well.*

Curriculum reviews revealed congruent findings where competencies, outcomes, content, assessment, and clinical practice durations were modified and standardized. Critical care, pain management, and postoperative care were the focus areas revision. Some programs also acknowledged efforts to standardize curriculum implementation using guiding documents developed by the local professional association. The course-based and less integrated curriculum is currently used in all but one institution, while an integrated curriculum has only recently been introduced.

### The exam improved in-school assessments

In this study, the most salient sub-category described by all study participants was the shift in in-school assessment practice to more impactful methods with better psychometric properties that direct and inspire students to work hard. Most respondents described an increased frequency and variety of exams and an established mechanism to review exam quality. A few FG participants also said that the feedback from the new in-school workplace-based assessments helped them demonstrate evidence-based practice. A graduate quoted:

Graduate 05: *We were assigned to work at my current facility in groups of five; all graduated and tested in the same year but from different institutions. We established a post-operative follow-up at the health facility. Also, all abdominal surgeries were previously performed under general anesthesia, but we have recently started using regional anesthesia. These improvements in clinical care result from incorporating and assessing those new skills into our training curriculum to prepare us for the licensing exam.*

During site visits, we noticed that the quality of written exams had improved noticeably as the focus turned to creating context-based items. We also observed workplace-based assessment tools and OSCE checklists preserved in files at most institutions.

Study participants, however, identified key assessment challenges, including increased faculty workload and the prevailing faculty propensity to use each assessment result to determine final course grades rather than leaving assessments ungraded and using them as opportunities for continuous performance improvement with feedback. Another challenge was ensuring that students did not become exam-oriented rather than aiming for comprehensive knowledge and acquiring clinical skills that encompass all the essential areas required for safe anesthetic care, whether assessed by the NLE or not.

### The exam driving the learning

Programs reported introducing various active learning methods such as special lectures, seminars with pre- and post-exams, case-based discussions, extended morning sessions, morbidity and mortality meetings, patient side sessions, and joint sessions with postgraduate students. Such practices appeared to vary among departments, with most changes reported in senior-year studies, where the licensing exam content is expected to focus.

Program Head 03: *In the past, neither the anesthesia students nor their department gave public health courses the attention they deserved. Because of the licensing exam, they are now paying particular attention to the area. The exam helped us implement the public health course as stated in the curriculum, with adequate emphasis on community practice.*

### The exam as a quality advocate

Most interviewees reported that the NLE served as a wheel to drive institutional quality improvement and assurance activities by establishing or strengthening units, committees, and coordinators. Programs also reported starting program self-reviews, despite gaps in regularity, thoroughness, and efforts to use review results for improvement.

Higher leader 03: *Efforts have been made to improve the quality of education. After the first [licensing] exam result was announced, we reorganized six quality assurance committees with the participation of different departments. We regularly monitor the work of each committee that helps initiate regular quality improvement tasks at our institution.*

Document reviews found consistent findings where all participating programs conducted at least two self-reviews using the national education program standard between 2017 and 2021. However, it is unjustifiable to attribute the number of reviews and performance changes solely to the licensing exam. From self-reviews, four of the ten main education standard areas showed notable improvement from 2017, each achieving more than 70% of the basic education standards: program goals and outcomes (A1), curriculum (A6), teaching-learning and assessment (A7), and student progress (A8). Although there was some progress in the remaining six standard areas, the percentages of basic standards met were still very low, requiring immediate attention.

### The wheels of the change process

Three of the six driving forces for educational changes were intrinsic, relating to faculty engagement in the NLE process, increased accountability, and improved collaboration. The remaining three were extrinsic forces related to feedback to institutions, the implementation of pilot OSCE, and COVID 19 and political instability (Table 3). Five of these six driving forces emerged as common sub-categories in both FG and interview transcript analyses, while the sixth, *faculty engagement in the NLE process*, emerged in the interview transcript.

**Table 3:** Summary of driving forces for educational changes and sample excerpts

Factor	Summary of the factor	Sample excerpts and document review
Feedback to teaching institutions (E,+)	Both groups of respondents considered feedback on the NLE performance of examinees by MOH as a critical positive driving force for the in-school changes by recognizing best-performing programs and informing areas of improvement.	Instructor 04: *This [feedback] helped us get recognition from the university. This acknowledgment results in financial, procedural, and other support… Besides, the feedback enabled us to examine our course delivery process.*
Introduction of pilot OSCE (E,+)	Following the pilot OSCE, most programs reportedly strengthened and effectively utilized their skill development labs (SDLs) by hiring skill lab assistants and expanding instructors’ duties to SDLs.	Graduate 02: *During the pilot, it was discovered that the practical exam’s content differed from what we were learning. At that point, all of the reserved skill lab items began to come out to assist us.*
COVID-19 and political instability (E,-)	Programs reported a temporary deviation from introducing quality enhancement interventions to quickly complete long-interrupted education due to the COVID-19 pandemic and national political instability. The measures taken to contain the COVID-19 infection impacted clinical education by drastically reducing student exposure.	Student 02: *Due to the current political unrest, we last had a morning session at our teaching institution over six months. The most common surgeries were casualties; I do not remember elective surgical cases.*
Faculty engagement in the NLE process (I,±)	Most interviewees perceived faculty engagement in different phases of the NLE as a positive driving force, preparing them to improve institutional assessment practices. However, those not engaged reported demotivation.	Program head 01: *Instructors who were not engaged in the [licensing] exam process perceived the institutional assessment changes as activities that should only be undertaken by those who were regularly involved.*
Increased accountability (I,+)	Interviewees, particularly from programs with lower student pass rates, reported increased accountability, forcing them to put in extra effort to prepare students for the NLE.	Higher leader 01: *The licensing examination increased our accountability as everyone was openly judged based on our program pass rate.*
Improved collaboration and networking (I,+)	The public notification of NLE pass rates made the exam an agenda item for institutional meetings. Better institutional pass rates were reported as embracing a positive image of programs, thereby enhancing inter-departmental collaboration. On document review, the NLE appeared as a discussion agenda in close to half of the academic committee minutes reviewed.	Instructor 07: *We contacted every department that offers courses for which we believe that the topic will appear on the licensing exam and informed them to pay attention to it when teaching.*

Note: Abbreviations: E, Extrinsic; I, Intrinsic. Note: Symbols: +, Positive driving force; -, Negative driving force; ±, Both positive & negative driving effects

### Perceptions about the licensing exam

All study participants expressed positive views about the purpose of a well-designed NLE. Notably, those interviewees who participated in the NLE process demonstrate strong ownership. On the other hand, some FG participants questioned the appropriateness of decisions made based on the NLE score in its current form:

*“How could it be justified to hold the student solely accountable [for failures] without fulfilling required teaching standards? For example, what I have learned and practiced differs significantly from what was asked on the [licensing] exam. The majority of the questions on the exam were impractical and less relevant to the anesthesia profession. Therefore, passing or failing this exam has nothing to do with my competence… I see the exam as a piece of paper that grants me a license rather than a measure of my skills”.* Graduate 14

## Discussion

Regulatory frameworks are essential to guarantee that the rapid expansions in health workforce education do not compromise the quality and safety of patient care, particularly when implementing task sharing and shifting [3, 8, 37]. Unfortunately, strong empirical evidence is very scarce and not enough to truly understand the impacts of NLEs, as a regulatory framework, on health profession education and patient care [12, 13, 16, 21, 26, 30]. In this study, we explored the experiences and perspectives of key stakeholders and conducted relevant document scrutiny regarding the educational effect of NLE and how the anesthesia programs modify their practices. Following the qualitative analysis of focus group and interview transcripts, twelve themes emerged, including six perceived educational changes and six driving forces.

A central finding of this study was that the NLE drives programs to introduce and strengthen a range of impactful contemporary assessment methods that inspire and direct student learning. Similar changes were reported in other studies, where NLE prompted the use of multiple in-school assessment methods to measure competency dimensions [19, 20, 38]. However, congruent formative assessments must precede the largely summative assessment changes identified in our study to enhance learning [20, 38, 39]. Also, setting up a mandatory assessment system can help programs make better decisions by coordinating and triangulating multiple individual assessments (summative and formative) across different content areas and learning domains [28, 39]. By establishing such a system, programs can lay the groundwork to address concerns about student exam-centeredness that may drive learning in an undesirable direction, particularly when the areas assessed in NLE do not reflect actual current practice.

Another prevailing finding of this study was the catalytic effect of the NLE on curriculum review and implementation. MOH feedback in the form of licensing exam data encouraged anesthesia programs to direct curriculum competencies, learning outcomes, and content to address the NLE priority areas using active learning methods. Our findings are consistent with those of other studies in which introducing a licensing exam or adopting a skill component contributed at least in part to driving curricular reforms [19, 20, 40, 41]. Designing the Ethiopian NLE based on the updated roles of graduates and blending it with some form of skill assessment can further enhance its educational effect. Otherwise, misalignment between what is taught in schools, what is practiced in hospitals, and what is assessed at the NLE will distract the NLE’s focus away from ensuring safe anesthetic care and may undermine the exam’s acceptance and validity.

Our study reported another essential impact of the NLE: hastening institutional quality improvement efforts through the meaningful engagement of academic leaders. This can be explained as an educational effect of the exam where licensing exam data serves as a standard to evaluate the performances of faculty, programs, and institutions and inform decisions to meet the teaching and social accountability duties [28, 38, 42]. In this regard, academic leaders’ steadfast commitment is critical to establishing a structure where NLE data can be effectively used to inform interventions [20]. Establishing such a responsive system requires a balanced, equitable and genuine collaboration on NLE activities between the regulator (MOH) and teaching institutions, thus ensuring that the exam matches the practice setting [43–45].

Our study participants reported that the NLE strengthened formal and informal interactions between students, teachers, and programs within an institution to better prepare students for the NLE. Though mainly confined to a few departments, this cooperation reportedly allowed the different departments to recognize each other’s contributions and establish recommended internal quality assurance systems. Besides, cross-institutional collaboration across anesthesia programs contributed to curriculum harmonization. A recent study by Sören Huwendiek et al. reported comparable findings where collaborations between stakeholders established the groundwork for sustainable educational reforms after licensing exams [40]. Another study reported that regional collaboration among schools was vital in establishing a standard assessment practice similar to NLEs [46]. As the Ethiopian NLE blended different contents, institutional collaboration across multiple departments should be enhanced to have a meaningful impact [47]. A more meaningful and sustainable network can be established by scaling existing collaborations using more structured, digitally-enhanced, and participatory models like the *‘hub and spokes’* and *‘community of practice’* [40, 48]. Similar East African anesthesia training institutions can be involved to form a regional alliance for better quality training.

The educational changes reported in this study happened predominantly in senior year studies, on which the licensing exam content is assumed to primarily focus on, with less attention being paid to juniors. Despite methodological variations and the duration of NLE implementation, NLE-driven changes were more widespread in other studies [18–20, 40]. One reason for this could be the differences in NLE models, with the Ethiopian single-step model aiming to cover all contents but with a predominant emphasis on anesthesia and critical care specialty areas. The discipline-based and ill-integrated curriculum used in all anesthesia teaching institutions, except one, may have also resulted in disparities in reforms across programs. Setting up program-level systems to coordinate departments and effectively implement the newly integrated anesthesia curriculum can help promote holistic reforms [18, 49, 50]. Also, establishing institutional qualification exam(s) bridging the basic science, clerkship, and anesthesia specialty years can help improve the breadth of changes. To sustain changes, programs will benefit from strengthening self-review and accreditation systems.

Finally, this study reported an improved program commitment to engage students and faculty in institutional learning and assessment practices. However, due to ineffective orientation and low student engagement in the NLE process, focus group participants expressed dissatisfaction with the NLE and believed that they alone were held responsible for exam failure – which challenged the exam’s acceptability. Similar findings were reported on a newly launched licensing exam, where a lack of effective communication with students and teaching institutions impacted the exam process [43]. Introducing a student-centered communication strategy by engaging student associations throughout the NLE process can improve acceptability.

### Strength and limitations

This research had several strengths. First, it involved all eligible anesthesia training institutions nationally. Second, data were gathered from students, graduates, different department instructors, program heads, and higher academic leaders, allowing for the assessment of change from various perspectives. Third, data triangulation was accomplished by analyzing relevant documents. Finally, data for this study was gathered using tools derived from a global standard and tailored to the Ethiopian setting, facilitating comparisons with other studies.

As opposed to longitudinal data collection at multiple points in time, the cross-sectional data collection we employed is an important limitation of the current study, as it makes it harder to examine and demonstrate causality between the NLE and its consequences. Another limitation is the inherent subjectivity of the study design and the difficulty of filtering the preconceptions and interests of the investigators and respondents to report positive or negative changes attributed to the NLE. Examining the specific elements in detail may provide a more in-depth understanding of the background than the overall educational changes explored in this study. Finally, although two investigators checked the coding and data analysis, no numeric intercoder agreement was computed.

## Conclusion

This qualitative study revealed that the Ethiopian NLE has prompted teaching institutions to improve their teaching-learning and assessment practices. Even though the NLE has only been in place for three years, it has begun to drive promising changes in curriculum content and delivery, faculty motivation and performance, and student behavior and learning approaches, at least partly in response to the exam. This study may have understated the extent of the changes since the COVID-19 pandemic and political unrest are likely to have thwarted some of the promising positive changes. Introducing and maintaining changes required unreserved dedication from academic leaders to take feedback from the MOH and put it into action. Accountability, engagement, and collaboration among key stakeholders were vital to ensure change sustainability.

While this study broke new ground regarding the impact of NLE in LMICs and non-physician training, it also has fundamental research implications. To have a holistic understanding of changes, future qualitative studies should attempt to explore the untoward consequences of implementing the NLE by incorporating the perspectives of students, instructors, and academic leaders. Besides, conducting a mixed-methods pre-post evaluation study on student outcome changes will provide a measurable estimate of graduates’ competence and corroborate arguments that NLE can result in improved quality of education. Perioperative standards of care and patient outcomes should be assessed to look into the association between the NLE score and clinical performance.

## Electronic supplementary material

Below is the link to the electronic supplementary material.

**Supplement 1:** Adherence to COnsolidated criteria for REporting Qualitative research (COREQ): 32 item Checklist

**Supplement 2:** Semi structured interview guide (English version)

**Supplement 3:** Focus group guide (English version)

## Data Availability

The datasets generated during and analyzed during the current study are not publicly available due to privacy and ethical concerns but are available from the corresponding author on reasonable request.

## Abbreviations

MOH: Ministry of Health
NLE: National Licensing Examination
OSCE: Objective Structured Clinical Examination
