# Supplementary material for: Impact of anesthetist licensing examination on quality of education in Ethiopia: a qualitative study of faculty and student perceptions
Source: BMC Med Educ. 2023 Jun 22;23:468. doi: 10.1186/s12909-023-04452-5 (PMC10288700; doi:10.1186/s12909-023-04452-5)
Supplement: Supplementary file 2 — Supplement 2: Semi structured interview guide (English version) [file 12909_2023_4452_MOESM2_ESM.pdf]

Tool 2: English version FG guide for students & graduates

CHANGES MADE TO ANESTHESIA EDUCATION PROGRAM AS A RESULT OF NLE

FOCUS GROUP STRUCTURE

Dear Colleagues,

I have indicated to you that I am supporting the Federal Ministry of Health to conduct a research project on the impacts of introducing a national licensing examination on the health workforce education and practice.

This focus group aims to obtain your ideas, experiences, and opinions regarding the changes made to the anesthesia education program as a direct result of introducing the national licensing examination.

The information obtained will only be used for research purposes and will remain confidential and your anonymity will remain protected at all times.

This focus group will take about 90 to 100 minutes to complete.

May I audio-record the discussion, as it would help me to listen to it again later and to make a transcript of the interview for data analysis purposes?

You have already read through the information booklet and listened to my presentation on it.

SECTION ONE: INTRODUCTION TO THE FOCUS GROUP

Instruction to the moderator (after receiving consent)

- Introduce yourself and provide a brief introduction about the study using the letter of information and the brief note indicated on the previous page
- Provide a brief explanation of how the focus group will be conducted, including the audio recording of conversations.
- Explain the following *ground rules* of focus groups

  Ground rules
- Inform the ground rules to participants by saying:

1. “As there are no wrong or right responses to discussion questions, all of your ideas and thoughts are respected and no criticism is allowed.”
2. “As you all have different backgrounds and experiences, please share your views even if they might differ from others.”
3. “Each one of you will have an equal opportunity to speak as I am interested in hearing the thoughts of you all. Thus, if any one of you is speaking too much, I may give the chance to others to speak. And, if you are not talking a lot, I may ask you to speak. The aim is to allow an individual of you to share your different unique experiences.”
4. “Please don't assume that you have to respond to me during our discussion. Feel free to respond to what your colleague has said, whether you agree or disagree, or if you want to give an example.”
5. I'm here to listen, ask questions, and ensure that everyone has an opportunity to speak.

- As part of the preparation for the focus group, ask participants to silence their cell phones.
- Moderator asks participants to introduce themselves by saying. “Now, let’s start. Let's go around the table one by one and learn more about each other. Tell us about your:
    1. Sex: ______________________
    2. Where do you work/ study _____________________________
    3. When did you take the NLE? (for graduates) ____________

SECTION TWO: MAIN FOCUS GROUP

Area	Focus group questions and probes	Time
Perception towards NLE (introduction)	1. How do you see the relevance of NLE in anesthesia education?<br>***Probes***:<br>▪ What do you think the purposes of NLE are?<br>▪ What do you think the advantages of NLE?	10 minutes
A1. Curriculum	2. What, if any, changes have been made to the competencies, content, and delivery of the anesthesia curriculum due to the NLE?<br>***Probes***:<br>▪ What changes have been made to the learning activities employed in the anesthesia program (any new method,)?	15 minutes

Area	Focus group questions and probes	Time
	▪ What elements (if any) of the curriculum content (basic sciences, public health, clinical science, and professional courses) have been changed due to NLE, and why?<br>▪ What changes in the teaching skills of instructors have been made?<br>▪ How well do you think the anesthesia curriculum (including learning activities) in your institution help students prepare for the NLE? How?<br>▪ Do you see yourself as a competent anesthetist after passing the NLE? Why?<br>3. How does the NLE affect the relationship between the different departments/ units involved in teaching the anesthesia students? (within the institution and across the nation)	
A2. Assessment	4. How does the introduction of the NLE impact the school's/ department's assessment policy?<br>***Probes***:<br>▪ Any modifications regarding the timing, number, and type of assessments per module/ course and curriculum at large?<br>▪ What has been changed regarding students' assessment to support their learning (formative) and decision (summative) in the classroom, simulated and clinical areas?	10 minutes
A3. Students	5. What, if any, has been changed in the student selection, admission, and support systems as a result of introducing the NLE?<br>***Probes***:<br>▪ How the number and quality of students is changed due to the NLE?<br>▪ What, if any, changes happened in your behaviors, motivation or study approaches as a student?<br>▪ How do the academic or social support changed due to the NLE?	15 minutes
A4. Academic staffs	6. What, if any, changes have been made on staff composition, role, and conduct?<br>***Probes***:<br>▪ Any change in the number and characteristics of academic and support staff?<br>▪ How does the NLE affect the role, conduct, and performance of anesthesia instructors (in planning, facilitating, assessing, and evaluating learning)?	10 minutes

Area	Focus group questions and probes	Time
A5. Educational resources	7. What, if any, changes have been made regarding the educational resources required for the classroom, skill lab, clinical area, and community-based teaching as a result of the NLE?	
A6. Quality assurance	8. What, if any, has been changed regarding the institutional quality assurance system as a direct result of introducing the NLE?<br>***Probes***:<br>▪ What changes have been made to improve the involvement of students in the QA process?<br>▪ What is changed on the emphasis given to QA (e.g. resource allocation, structural modification, etc.)	10 minutes
A7. Governance and administration	9. How does the introduction of the NLE affect institutional governance and administration?<br>***Probes***:<br>▪ What has been changed on the involvement of students in decision-making regarding departmental issues?<br>▪ What has been changed regarding the administrative structure and support to the department?	10 minutes
Way forward	10. What do you want to see changed about NLE?<br>11. Overall, how do you judge the effectiveness of the NLE in assessing the competence of anesthesia graduates?	10 minutes

I would like to thank you for your time and participation in this study.
