# Supplementary material for: Impact of anesthetist licensing examination on quality of education in Ethiopia: a qualitative study of faculty and student perceptions
Source: BMC Med Educ. 2023 Jun 22;23:468. doi: 10.1186/s12909-023-04452-5 (PMC10288700; doi:10.1186/s12909-023-04452-5)
Supplement: Supplementary file 3 — Supplement 3: Focus group guide (English version) [file 12909_2023_4452_MOESM3_ESM.pdf]

Tool 1: English version interview guide

CHANGES MADE TO ANESTHESIA EDUCATION PROGRAM AS A RESULT OF NLE

INTERVIEW STRUCTURE

Dear Colleague,

I have indicated to you that I am supporting the Federal Ministry of Health to conduct a research project on the impacts of introducing a national licensing examination on the health workforce education and practice.

This interview aims to obtain your ideas, experiences, and opinions regarding the changes made to the anesthesia education program as a direct result of introducing the national licensing examination.

The information obtained will only be used for research purposes and will remain confidential and your anonymity will remain protected at all times.

This interview will take about 30 to 60 minutes to complete.

Do you have any questions before we start the interview?

May I audio-record the interview, as it would help me to listen to it again later and to make a transcript of the interview for data analysis purposes?

You have already read through the information booklet and listened to my presentation on it.

SECTION ONE: BASIC INFORMATION

I would like to start with some basic demographic questions about your self

1. Sex: _______________________
2. Profession: _____________________
3. Name of institution: _______________________
4. Department (if applicable): ______________
5. Current position: ____________________
6. How long have you been working in this teaching institution? ____________
7. How long have your school been engaged in the NLE? ____________

SECTION TWO: MAIN INTERVIEW

<table>
<tr><th>Area</th><th>Interview Questions and Probes</th></tr>
<tr><td>A1. Curriculum</td><td>1. What, if any, changes have been made to the competencies, content, and delivery of the anesthesia curriculum due to the NLE and how this affect the quality of anesthesia education?

<u>Probes</u>:
▪ What changes have been made to the anesthesia program competencies and outcomes?
▪ What elements (if any) of the basic sciences have been changed (added or omitted) and why?
▪ What elements (if any) of the social and population health have been changed (added or omitted) and why?
▪ What elements (if any) of the clinical sciences have been changed (added or omitted) and why?
▪ What elements (if any) of the professional courses/ modules have been changed (added or omitted) and why?
▪ Any new learning method introduced/ omitted?
▪ Any shift in the principle of selecting teaching methods?
▪ Changes in teaching skills of instructors?
▪ Do you believe the anesthesia curriculum (including learning activities) in your institution help students prepare for the NLE?

2. How does the NLE affect the relationship between the different departments/ units involved in teaching the anesthesia students?</td></tr>
<tr><td>A2. Assessment</td><td>3. How does the introduction of the NLE impact the school's/ department's assessment policy?

<u>Probes</u>:
▪ Any modifications regarding the timing, number, and type of assessments per module/ course and curriculum at large?
▪ How are the new assessments integrated into the curriculum?

4. What, if any, changes have been made due to the NLE regarding students' assessment to support their learning (formative) and decision (summative) in the classroom, simulated and clinical areas?

<u>Probes</u>:
▪ How assessments are planned (e.g. blueprint)?</td></tr>
</table>

Area	Interview Questions and Probes
	▪ What new formative methods are introduced?<br>▪ What new summative methods are introduced<br>▪ What has been changed on pass marks & the way they are set?<br>▪ What is new regarding student support after assessments?<br>▪ What measures are introduced to improve the quality and impacts of in-school exams (quality assurance)?
A3. Students	5. What, if any, has been changed in the student selection, admission, and support systems as a result of introducing the NLE? How does this affect the quality of anesthesia education?<br>***Probes***:<br>▪ How the number and quality of students is determined & any changes made?<br>▪ What, if any, changed in student behaviors, motivation or study approaches?<br>▪ What has been changed to improve diversity?<br>▪ How does the academic or social supports change due to the NLE?
A4. Academic staffs	6. What, if any, changes have been made on staff composition, role, and conduct?<br>***Probes***:<br>▪ Any change in the number and characteristics of academic and support staff?<br>▪ Any change in the performance of instructors (in planning, facilitating, assessing, and evaluating learning)?<br>▪ How does the NLE affect the role and conduct of anesthesia instructors?<br>▪ What, if any, new instructors-related policies (e.g. staff recruitment, development, preparation, and evaluation) are introduced in response to the NLE in your institution?
A5. Educational resources	7. What, if any, changes have been made regarding the educational resources as a result of the NLE?<br>***Probes***:<br>▪ Changes made regarding classroom resources, including IT?

Area	Interview Questions and Probes
	▪ Changes made on resources required for community-based teaching?<br>▪ Changes made regarding clinical teaching resources?
A6. Quality assurance	8. What, if any, has been changed regarding the institutional quality assurance system as a direct result of introducing the NLE?<br>***Probes***:<br>▪ What changes have been made to improve the involvement of different stakeholders in the QA process?<br>▪ What is changed on the emphasis given to QA (e.g. resource allocation, structural modification, etc.)
A7. Governance and administration	9. How does the introduction of the NLE affect institutional governance and administration?<br>***Probes***:<br>▪ Do you think the national examination is beneficial for your institution? How?<br>▪ What governance changes have been made to review and strengthen the performance of the anesthesia department/ school?<br>▪ What has been changed on budget allocation to the department?<br>▪ What has been changed on the involvement of students and instructors in decision-making regarding departmental issues?<br>▪ What has been changed regarding the administrative structure and support to the department?
Way forward	10. What do you want to see changed about NLE in the future?<br>11. In the next five years, how do you see your institution progressing?

I would like to thank you for your time and participation in this study.
